# An Improved Proxy Re-Encryption Scheme for IoT-Based Data Outsourcing Services in Clouds

**DOI:** 10.3390/s21010067

**Published:** 2020-12-24

**Authors:** Han-Yu Lin, Yao-Min Hung

**Affiliations:** Department of Computer Science and Engineering, National Taiwan Ocean University, Keelung 202, Taiwan; n7773246@gmail.com

**Keywords:** IoT, data outsourcing, proxy re-encryption, cloud computing, bilinear pairing

## Abstract

IoT-based data outsourcing services in clouds could be regarded as a new trend in recent years, as they could reduce the hardware and software cost for enterprises and obtain higher flexibility. To securely transfer an encrypted message in the cloud, a so-called proxy re-encryption scheme is a better alternative. In such schemes, a ciphertext designated for a data aggregation is able to be re-encrypted as one designated for another by a semi-trusted proxy without decryption. In this paper, we introduce a secure proxy re-encryption protocol for IoT-based data outsourcing services in clouds. The proposed scheme is provably secure assuming the hardness of the bilinear inverse Diffie–Hellman problem (BIDHP). In particular, our scheme is bidirectional and supports the functionality of multi-hop, which allows an uploaded ciphertext to be transformed into a different one multiple times. The ciphertext length of our method is independent of the number of involved IoT nodes. Specifically, the re-encryption process only takes one exponentiation computation which is around 54 ms when sharing the data with 100 IoT devices. For each IoT node, the decryption process only requires two exponentiation computations. When compared with a related protocol presented by Kim and Lee, the proposed one also exhibits lower computational costs.

## 1. Introduction

The research of IoT-based applications [1] has received much attention recently. The concept of IoT-based cloud computing has also revolutionized people’s lives. It allows IoT devices to remotely connect to cloud servers for requesting various cloud services. These IoT devices can also upload sensed data to the cloud for sharing, processing, and storing. Using IoT sensor technologies, we can realize the notion of connecting all real world things to the Internet without the intervention of human beings. For example, in supply chain networks, a factory could use Radio Frequency Identification (RFID) tags [2] to calculate the product count and trace the current location of shipped products. According to the definition of the European Telecommunications Standards Institute (ETSI) [3], the structure of IoT can be divided into three layers, including the lower perception layer, the middle network layer, and the upper application layer. The perception layer utilizes the techniques of embedded systems, RFID, and wireless sensor networks (WSNs) to collect data [4]. The network layer is responsible for receiving data of the perception layer and then forwards them to the application layer, which mainly employs all kinds of telecommunication techniques such as Bluetooth, WiFi, WiMax, etc. [5]. The application layer combines received data with practical development techniques to provide integrated IoT services [6]. Generally speaking, there are four communication modes for IoT devices [7]:

(i) **Device-to-Device Communication:** Two or more devices could communicate with each other without relying on middleware servers. These devices can work in various networks such as Bluetooth, Z-Wave, and ZigBee, etc.
(ii) **Device-to-Cloud Communication:** IoT devices could directly connect to cloud service providers for exchanging information and controlling flow messages. Such a communication mode usually utilizes existing communication mechanisms like Ethernet or WiFi.
(iii) **Device-to-Gateway Communication:** IoT devices connect to the gateway to obtain cloud services. Specifically, application software of the gateway will provide the security and functionality for data and protocol conversion.
(iv) **Back-End Data-Sharing Model:** It allows users to combine the data output of other sources and analyze the intelligent object data of cloud services. It thus could be regarded as an extension of device-to-cloud communications.

Up to the present, IoT has become a popular term emphasizing the ability to extend the connectability of the Internet for combining various objects, sensors, terminal devices, and even facilities [3]. In recent years, cloud outsourcing services have played an important role in enterprises, since they could reduce more hardware and software costs. However, cloud data are not always safe due to various security concerns and untrusted cloud servers. To increase the security of cloud outsourcing services, a suitable cryptographic protocol is essential.

Traditionally, a symmetric or asymmetric encryption scheme only enables an intended recipient to decrypt the ciphertext during the entire communication process. The cryptographic scheme of proxy re-encryption (PRE) [8] further allows a ciphertext designated for some entity to be securely transformed into the one designated for another by a semi-trusted entity called a proxy. In such a scheme, a device receiving a ciphertext can utilize its private key to decrypt it and the proxy responsible for transforming the ciphertext learns nothing about the original plaintext.

When a ciphertext is able to be transformed multiple times, we refer to this property as multi-hop. On the contrary, a single-hop PRE only allows a ciphertext to be transformed once. In practice, a PRE scheme has lots of applications [9,10,11,12] in the real world such as forwarding of confidential e-mails, key escrow, key distribution, etc.

### Contributions

For facilitating the gradually popular IoT-based data outsourcing services in cloud environments, in this work, the authors propose a new PRE scheme. The proposed scheme could be applied in practical applications such as smart factory management and IoT-based healthcare services, in which a patient sensor could send the encrypted data to a proxy server for sharing with other devices. Some concrete contributions of our mechanism are stated as follows:

(i) The proposed PRE scheme is bidirectional and supports the functionality of multi-hop.
(ii) Our scheme is provably secure in the random oracle model using the Bilinear Inverse Diffie–Hellman assumption.
(iii) The ciphertext size of our scheme is constant, i.e., it is independent of the number of IoT nodes.
(iv) The re-encryption process only takes one exponentiation computation.
(v) The proposed PRE scheme earns more computational savings compared with a related work presented by Kim and Lee.

The remaining parts of this work would be arranged as follows. In the next section, we describe essential research backgrounds. Our proposed PRE system is fully presented in Section 3. In Section 4, we discuss its security and demonstrate comparisons with a related method. Lastly, a conclusion is given in Section 5.

## 2. Research Backgrounds

### 2.1. Related Works

For sharing private multimedia content in social cloud storage, Wang et al. [13] proposed a non-transferable unidirectional PRE scheme. The non-transferable property of PRE schemes could prevent a malicious proxy from further re-delegating the decryption rights to other users, so as to protect the privacy of the data owner’s data.

For secure communication between IoT devices and the gateway, Henriques and Vernekar [14] proposed a hybrid approach by combining symmetric and asymmetric cryptographic techniques. Especially, they utilized random keys generated from system timestamps to deal with the problem of session key distribution of symmetric algorithms. Their mechanism implements the modified Vigenere Cipher and the RSA algorithm.

Considering the functionality of multi-hop, Li et al. [15] introduced a multi-hop homomorphic identity-based PRE mechanism via branching program. Different from most existing PRE protocols that are mainly based on the Diffie–Hellman assumption, their scheme is based on the decisional learning with errors (LWEs) assumption. The construction of their work is modified from a lattice-based identity-based encryption (IBE) scheme. That is to say, they realized the idea of assembling a lattice-based PRE from a lattice-based IBE. Moreover, they also showed that their approach supports homomorphic evaluation.

Thinking of the merits of identity-based algorithms, Wang et al. [12] presented an ID-based PRE scheme (called IBPRE+) for secure cloud data sharing. In their scheme, the data owner could utilize a random number of encryption processes to dynamically control the capability of sharing. In 2016, without utilizing random oracles, Ge et al. [16] presented a key-policy attribute-based PRE scheme whose security relies on the 3-weak decisional bilinear Diffie–Hellman inversion (3-wDBDHI) assumption. Later, Fugkeaw and Sato [17] proposed a lightweight PRE scheme supporting mobile revocation management in cloud computing. Specifically, their mechanism could provide the functionalities of re-encryption key generation, re-encryption key update, and re-encryption key renewal.

In 2017, Chandu et al. [18] designed and implemented a hybrid encryption for ensuring the transmission security of IoT data. They employed the software of Xilinx ISE-Design 14.5 and Xilinx SPARTAN-6 to implement the proposed design. However, their method did not support the functionality of re-encryption.

Some researchers [19,20] further combined PRE mechanisms with the property of keyword search. That is to say, a user can choose a keyword and request its corresponding ciphertext. Liang et al. [21] also combined attribute-based encryption with the PRE scheme.

In 2016, Akhil et al. [22] applied the PRE scheme to QR code security and thus can provide efficient and secure data transmission between the sender and the receiver. In 2018, Zeng and Choo [23] introduced a conditional PRE scheme which could be utilized in secure cloud storage. They also demonstrated that their construction has lower computational costs and smaller ciphertext size. Hussain et al. [24] utilized binary-bit sequence and the XOR operation to design an encryption scheme for IoT communication. In their scheme, the data will be encrypted at multiple stages and the required encryption time is shorter than the traditional RSA system.

In 2019, Krishnamoorthy et al. [25] used near ring to introduce a privacy-preserving PRE scheme for IoT security. They analyzed the security of their scheme under equivalent private key attacks and chosen plaintext attacks. Although they claimed that their scheme allows IoT devices to efficiently store and manage their sensitive data, there is no related performance evaluation or comparisons presented in their work.

In 2020, Fan et al. [26] proposed a key-aggregate PRE with dynamic condition generation by multilinear map. A significant property of their scheme is that the size of public parameters remains small when the number of re-encryption keys becomes large. This allows a data owner to directly share his/her encrypted data on the cloud with the assistance of a proxy. Up to present, many PRE variants [27,28,29,30,31,32,33,34] have been proposed.

When PRE schemes are applied in IoT-based applications such as remote healthcare [35] and smart factory management, computation complexity is the biggest concern. Since previous protocols might not be suitable for IoT-based PRE platforms, in 2018, Kim and Lee [36] proposed a new PRE scheme for guaranteeing IoT device security. They claimed that their scheme could significantly reduce the cost of re-encryption. However, they failed to provide provable security and the overall computation complexity of their mechanism is still too high. Motivated by the above reason, the authors will devote themselves to the design and construction of a more efficient and secure PRE scheme for IoT-based application environments.

### 2.2. Preliminaries

A PRE scheme is one of the public key mechanisms whose security mainly rely on intractable computation problems such as the factorization and discrete logarithm problems. The former uses a composite number as the modulus while the latter employs a prime number as the modulus. In the proposed scheme, we will adopt the bilinear pairing operation from elliptic curves. For facilitating interested readers with better understanding of the proposed work, we first recall the definition of bilinear pairing as follows:

Definition of Bilinear Pairing:

Let *q* be a large prime and (***G***_1_, ***G***_2_) be two multiplicative groups of the order *q*. The symbol *e*: ***G***_1_ × ***G***_1_ → ***G***_2_ is defined as a bilinear map having some properties below:

(i) Bilinearity:
  Given two group elements in ***G***_1_, say *g*_1_ and *g*_2_, and two integers in *Z_q_*, say *a* and *b*, the value *e*(*g*_1_*^a^*, *g*_2_*^b^*) is equivalent to the value *e*(*g*_1_, *g*_2_)*^ab^*.
(ii) Non-degeneracy:
  There are two group elements in ***G***_1_, say *g*_1_ and *g*_2_ such that *e*(*g*_1_, *g*_2_) ≠ 1.
(iii) Computability:
  Given two group elements in ***G***_1_, say *g*_1_ and *g*_2_, there exists an efficient algorithm which could derive the value *e*(*g*_1_, *g*_2_).

When we design and implement a cryptographic protocol in pairing-based systems, the computational problem such as the bilinear Diffie–Hellman problem or its related variant, i.e., the bilinear inverse Diffie–Hellman problem, is commonly adopted. The two problems are polynomial time reducible, i.e., they are regarded as equivalent. We describe these problems and their corresponding assumptions below:

(i) ***Bilinear Diffie–Hellman Problem (BDHP)***
  Given elements *g*, *g^a^*, *g^b^*, *g^c^* ∈ ***G***_1_ for three positive integers *a*, *b*, *c* ∈ Z_q_^*^, the Bilinear Diffie–Hellman problem is to derive the ***G***_2_ element *e*(*g*, *g*)*^abc^*.
(ii) ***Assumption of Bilinear Diffie–Hellman (BDH)***
  The assumption of BDH states that the probability for any probabilistic polynomial time algorithm A to successfully break the BDHP is negligible.
(iii) ***Bilinear Inverse Diffie–Hellman Problem (BIDHP)***
  Given elements *g*, *g^a^*, *g^b^* ∈ ***G***_1_ for some positive integers *a*, *b* ∈ Z_q_^*^, the BIDHP is to compute the value *e*(*g*, *g*)*^a^*^^−1^^*^b^*∈ ***G***_2_.
(iv) ***Assumption of Bilinear Inverse Diffie–Hellman (BIDH)***
  The assumption of BIDH states that the probability for any probabilistic polynomial time algorithm A to successfully break the BIDHP is negligible.

## 3. Proposed PRE Scheme

**Figure 1:**
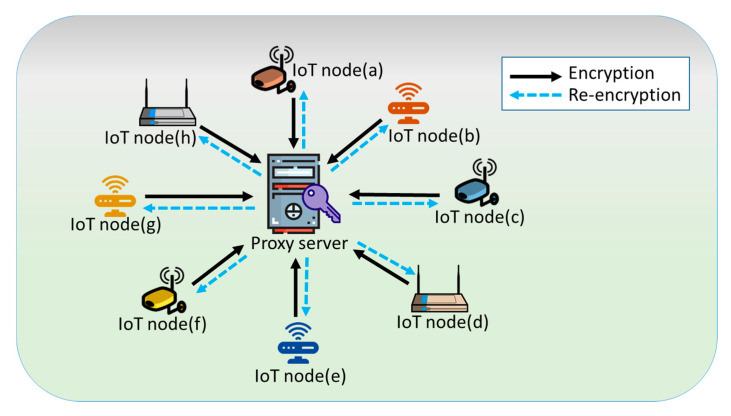
The idea of IoT-based data outsourcing services using proxy re-encryption (PRE).

The idea of the proposed PRE scheme is illustrated as Figure 1, in which an IoT node could share its encrypted data among various devices with the assistance of a proxy server. We present the proposed PRE scheme in detail. The composed algorithms are first defined. Then, a substantial construction is given.

### 3.1. Algorithms

Without loss of generality, we could divide the proposed PRE scheme into six algorithms, i.e., Initialize, KeypairGen, ReEnKGen, Encrypt, ReEncrypt, and Decrypt. The purpose of each algorithm is described as follows:

The Initialize(1*^k^*) algorithm is used for generating system public parameters by inputting a security parameter *k*. Given a user index *i*, the KeypairGen(*i*) algorithm could create a corresponding key pair including a private key and a public one. Additionally, given two private keys, say *x_i_* and *x_j_*, the ReEnKGen(*x_i_*, *x_j_*) algorithm computes the corresponding re-encryption key, say *rk_i_*_→_*_j_*, which transforms a ciphertext originally decrypted by the private key *x_i_* into the one decrypted by the private key *x_j_*. In the Encrypt(*Y_i_*, *m*) algorithm, given a public key *Y_i_* and a message *m*, the algorithm encrypts *m* with *Y_i_* and returns the final ciphertext. Similarly, in the ReEncrypt(*rk_i_*_→_*_j_*, *δ_i_*) algorithm, given a designated re-encryption key *rk_i_*_→_*_j_* along with a ciphertext, say *δ_i_*, the algorithm transforms the latter into a new ciphertext *δ_j_* that is encrypted with the public key *Y_i_* by the assistance of the former. Lastly, given a designated private key *x_j_* along with a ciphertext *δ_j_*, the Decrypt(*x_j_*, *δ_j_*) algorithm decrypts *δ_j_* with *x_j_* and then, returns either a decrypted message *m* or an invalid symbol ⊥.

### 3.2. Construction

According to the above algorithms, we present a substantial formation based on bilinear pairings as follows. Some used parameters are defined as in Table 1.

By taking a security parameter *k* as the input, the Initialize(1*^k^*) algorithm will determine two multiplicative groups (***G***_1_, ***G***_2_) whose order is a prime number *q*. The symbol *g* denotes a generator of ***G***_1_ and *e* is the operation of bilinear pairing satisfying that *e*: ***G***_1_ × ***G***_1_ → ***G***_2_. There are also two secure one-way hash functions, i.e., *h*_1_: {0, 1}*^k^* → {0, 1}*^k^* and *h*_2_: ***G***_2_ → Z_q_^*^. Finally, the algorithm will publish system parameters *params* which includes {***G***_1_, ***G***_2_, *e*, *g*, *q*, *h*_1_, *h*_2_}.

Given an arbitrary index *i* as the input, the KeypairGen(*i*) algorithm randomly chooses an integer *x_i_* ∈ *Z_q_* as the private key and then, computes *Y_i_* = *g^x^_^i^_* as the corresponding public key. To generate a re-encryption key, the ReEnKGen(*x_i_*, *x_j_*) algorithm first takes the input of two private keys, say *x_i_* and *x_j_*, and then, computes *rk_i_*_→_*_j_* = *x_j_*/*x_i_* mod *q* as a re-encryption key for subsequent re-encryption processes.

To produce a ciphertext *δ_i_*, the Encrypt(*Y_i_*, *m*) algorithm taking the input of a designated public key *Y_i_* and an arbitrary message *m*, where |*m*| = *k*_0_ first picks a random number *z* ∈ {0, 1}*^k^*_^1^_ in which *k*_1_ = *k* − *k*_0_ and then, computes

*c* = *g^h^*_^1^_^(*m* || *z*)^, (1)

*a* = *e*(*c^z^*, *Y_i_*), (2)

*b* = (*m* || *z*)*h*_2_(*e*(*c*, *g^z^*)) mod *q* (3)

Here, *δ_i_* is viewed as the resulted ciphertext composed of *a*, *b*, and *c*. In the re-encryption phase, given a ciphertext *δ_i_* = (*a*, *b*, *c*) which is encrypted by the public key *Y_i_* along with a re-encryption key, say *rk_i_*_→_*_j_*, the ReEncrypt(*rk_i_*_→_*_j_*, *δ_i_*) algorithm re-encrypts the ciphertext *δ_i_* by computing

(4) $$a^{\prime }=a^{rk_{i\to j}}\;(=\;e({Y_{j}}^{z},\;c))$$

Lastly, the re-encrypted ciphertext is *δ_j_* which is composed of *a*′, *b*, and *c*. To decrypt a ciphertext *δ_j_* = (*a*′, *b*, *c*) (which is encrypted by the public key *Y_j_*) and an intended private key *x_j_*, the Decrypt(*x_j_*, *δ_j_*) algorithm first performs the decryption process by computing

(5) $$m||z=b\cdot {ℎ}_{2}{\left(a^{\prime x_{j}{}^{-1}}\right)}^{-1}$$

and then verifies whether the equality *c* = *g^h^*_^1^_^(*m* || *z*)^ holds. If it does, the algorithm will output *m*; otherwise, an error symbol ⊥ is returned instead.

The correctness of Equation (5) could be easily checked as follows. Derived from Equation (5), we obtain

$$b\cdot {ℎ}_{2}{\left(a^{\prime x_{j}{}^{-1}}\right)}^{-1}=(m||z){ℎ}_{2}\left(e\left(g^{z},c\right)\right){ℎ}_{2}{\left(e{\left(Y_{j}{}^{z},c\right)}^{x_{j}{}^{-1}}\right)}^{-1}=(m||z){ℎ}_{2}\left(e\left(g^{z},c\right)\right){ℎ}_{2}{\left(e\left(g^{z},c\right)\right)}^{-1}=m||z$$

(by Equations (3) and (4)), which verifies the correctness of Equation (5).

## 4. Security Analysis and Performance

To analyze the security of the proposed PRE scheme, a defined security model of confidentiality is first given in Section 4.1 and then, the authors will adopt it to prove the proposed mechanism using the security proof model of random oracles in Section 4.2. Additionally, the performance evaluation will also be conducted in Section 4.3.

### 4.1. Security Model

The crucial security requirement of PRE schemes is confidentiality, i.e., a secure PRE scheme should be able to withstand the attack of adaptively chosen ciphertext (abbreviated as CCA). We state such CCA security model in relation to our proposed PRE scheme as follows:

**Definition 3. (CCA Security)** We say that a PRE scheme is unconditionally secure under CCA provided that there is no probabilistic adversary A who runs in polynomial time and has the non-negligible advantage to win the following simulation game in which an algorithm B behaves as a challenger:

**Setup:** At first, B calls the algorithm of Initialize(1*^k^*) to create public parameters, say *params*, which will be forwarded to A.

**Phase 1:** The adversary A is allowed to adaptively submit the following queries:

The first KeypairGen(*i*) query can be further classified into uncorrupted and corrupted. In the former case, B will return the public key *Y_i_* to A by calling the KeypairGen(*i*) algorithm. In the latter case, B also sends the private key *x_i_* to A. In the ReEnKGen(*Y_i_*, *Y_j_*) query, B will return a re-encryption key *rk_i_*_→*j*_ to A by calling the ReEnKGen(*x_i_*, *x_j_*) algorithm. It is compulsory that the indexes (*i*, *j*) should be uncorrupted or corrupted. When A makes the ReEncrypt(*Y_i_*, *Y_j_*, *δ_i_*) query in which the public keys (*Y_i_*, *Y_j_*) are created by previous KeypairGen queries, B would return a re-encrypted ciphertext *δ_j_* by calling the ReEncrypt(*rk_i_*_→*j*_, *δ_i_*) algorithm. Moreover, when A makes the Decrypt(*Y_j_*, *δ_j_*) query in which the public key *Y_j_* is generated by a previous KeypairGen query, B would return either a message or an error symbol by calling the Decrypt(*x_j_*, *δ_j_*) algorithm.

**Challenge:** The adversary A selects an uncorrupted public key Y* along with two fixed-length messages (*m*_0_, *m*_1_). The challenger B will compute a ciphertext δ* of the message *m_λ_*, where *λ* is randomly picked from {0, 1} and then, send the challenge δ* to A.

**Phase 2:** The adversary A could continue to submit queries below:

In this phase, the index of any corrupted KeypairGen query could not be the target challenge Y* or any derivative such as Y**. The ReEnKGen(*Y_i_*, *Y_j_*) query is the same as Phase 1. Similarly, the ReEncrypt(*Y_i_*, *Y_j_*, *δ_i_*) query is the same as Phase 1 except that an error symbol might be returned, provided that the public key *Y_j_* is corrupted and *δ_i_* is one derivative of the target challenge δ*. The Decrypt(*Y_j_*, *δ_j_*) query is also the same as Phase 1 except that an error symbol might be returned, provided that *δ_j_* is one derivative of the target challenge δ*.

**Guess:** Lastly, a guessed bit *λ*′ will be outputted by the adversary A. We say that A is the winner of the above game as long as *λ*′ = *λ*. Specifically, the advantage of A could be defined as *Adv*(A) = |Pr[*λ*′ = *λ*] *−* 1/2|.

### 4.2. Security Analysis

Following the previous security model of CCA, the authors demonstrate that the constructed PRE protocol is secure using the proof model of random oracles.

**Theorem 1. (Confidentiality)** In the security proof model of random oracles, the proposed PRE scheme could resist the attacks of the adaptively chosen ciphertext (CCA), provided that there exists no probabilistic adversary running in polynomial time and having the non-negligible advantage to break the intractable BIDHP.

**Figure 2:**
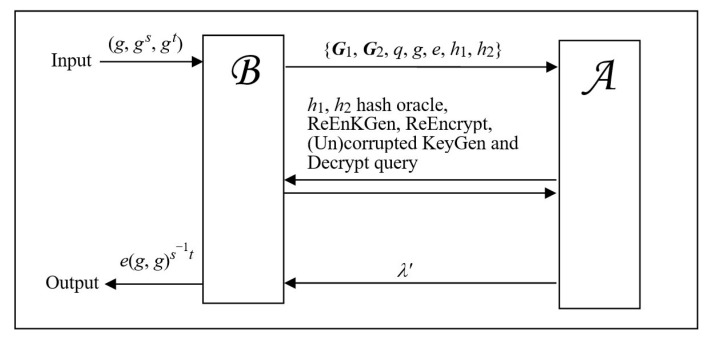
The proof idea of confidentiality in Theorem 1.

**Proof:** This proof idea of confidentiality is illustrated in Figure 2. We would complete this security proof by showing that a BIDHP breaker called B could be built by calling a probabilistic PRE adversary A whose running time is polynomial, say *t*, and has the non-negligible advantage *ε* to defeat the constructed mechanism under CCA attacks. We define the maximum times of each allowed query that A could make below.

*n_h_*__1__: #maximum *h*_1_ hash oracles;

*n_h_*__2__: #maximum *h*_2_ hash oracles;

*n_ckg_*: #maximum corrupted KeypairGen queries;

*n_ukg_*: #maximum uncorrupted KeypairGen queries;

*n_rkg_*: #maximum ReEnKGen queries;

*n_ren_*: #maximum ReEncrypt queries;

*n_dec_*: #maximum Decrypt queries.

The BIDHP breaker B is responsible for answering the queries made by A. Its objective is to compute *e*(*g*, *g*)*^s^*^^−1^^*^t^* from given input values (*g*, *g^s^*, *g^t^*).

**Setup:** At first, B calls the algorithm of Initialize(1*^k^*) for creating public parameters *params* = {***G***_1_, ***G***_2_, *e*, *g*, *q*, *h*_1_, *h*_2_} and forwards them to A.

**Phase 1:** By definition, A is able to adaptively submit the following queries:

To answer an *h*_1_(*m* || *z*) hash query, B maintains an *h*_1_-table storing (*m* || *z*, *v*_1_), in which *m* || *z* is submitted by A and *v*_1_ ∈ {0, 1}*^k^* is the return value randomly chosen by B. To answer an *h*_2_(*W*) *hash* query, B maintains an *h*_2_-table storing (*W*, *v*_2_) in which *W* is submitted by A and *v*_2_ ∈ Z_q_^*^ is the return value randomly chosen by B. To answer an uncorrupted KeypairGen(*i*) query, B maintains a Ukey-table storing (*x_i_*, *Y_i_*), in which *x_i_* is randomly chosen from *Z_q_* and the return value *Y_i_* is computed as (*g^s^*)*^x^_^i^_*. To answer a corrupted KeypairGen(*j*) query, B maintains a Ckey-table storing the return values (*x_j_*, *Y_j_*), in which *x_j_* is randomly chosen from *Z_q_* and *Y_j_* is computed as *g* *^x^^j^*. To answer an ReEnKGen(*Y_i_*, *Y_j_*) query, B first checks if both of (*Y_i_*, *Y_j_*) is in either the Ukey-table or the Ckey-table. If it does, the return value *rk_i_*_→*j*_ is computed as *x_j_*/*x_i_* mod *q*. To answer a ReEncrypt(*Y_i_*, *Y_j_*, *δ_i_*) query, B first checks whether both of (*Y_i_*, *Y_j_*) is in either the Ukey-table or the Ckey-table. If it does, the returned ciphertext *δ_j_* is computed as (*a*′, *b*, *c*) in which *a*′ = *a^x^_^j^_*^/*x*^*_^i^_* (= *e*(*Y_j_^z^*, *c*)). If it does not, B finds out a matched record (*m* || *z*, *v*_1_) in which *c* = *g^v^*_^1^_ and *a* = *e*(*Y_i_^z^*, *c*) from the *h*_1_-table and then, the returned partial ciphertext *a*′ would be computed as *e*(*Y_j_^z^*, *c*). Still, when no such records exist, B would return an error symbol ⊥. Finally, in a Decrypt(*Y_j_*, *δ_j_*) query, if *Y_j_* is in the Ckey-table, B directly calls the Decrypt(*x_j_*, *δ_j_*) algorithm and returns the result. If not, B finds out a matched record (*m* || *z*, *v*_1_) in which *c* = *g^v^*_^1^_, *a* = *e*(*Y_j_* *^z^*, *c*) and *b* = *h*_2_(*e*(*g^z^*, *c*))(*m* || *z*) from the *h*_1_-table and then outputs *m*. Nevertheless, if there exists no such records, the symbol ⊥ indicating an error would be outputted.

**Challenge:** The adversary A selects an uncorrupted public key Y* along with two fixed-length messages (*m*_0_, *m*_1_). The challenger B will compute a ciphertext δ* of the message *m_λ_* where *λ* is randomly picked from {0, 1} by the processes below:

1. Search the maintained Ukey-table for a record (x*, Y*);
2. Randomly select z* and b* from {0, 1}^*k*_1_^ and Z_q_^*^, respectively;
3. Compute a* = *e*(g^x^^* z*^, c*) and let c* be *g^t^*, i.e., this parameter *t* is implicitly set to be the output of *h*_1_(*m_λ_* || z*) and the value b*(*m_λ_* || z*)^−1^ is implicitly defined as the output of ${ℎ}_{2}\left(a^{*{\left(sx^{*}\right)}^{-1}}\right)$;

Here, the challenge ciphertext δ* is composed of (a*, b*, c*).

**Phase 2:** After obtaining the challenge, A could continue submitting queries as described in Definition 3.

**Guess:** The adversary A returns the output of a bit *λ*′.

**Output:** The BIDHP breaker B computes the answer as $W^{z^{*-1}}$, in which *W* is randomly chosen from the *h*_2_-table.

**Simulation Analysis:** According to the above simulation processes, some queries might return false results if the necessary precondition is not fulfilled. To evaluate the advantage of the constructed breaker B, we first define several probability events below.

SIP: the simulation is perfectly finished;

ReEnc_Err: an error occurs during a ReEncrypt query;

Dec_Err: an error occurs during a Decrypt query;

QH: an *h*_1_(*m_λ_* || z*) oracle is queried in phase 2.

An error occurring in either a ReEncrypt or a Decrypt query is mainly due to the fact that no matched records are kept in the *h*_1_-table—that is, the adversary A has derived/guessed the correct return value with respect to an *h*_1_ hash oracle. The probability of this condition is not greater than 1/2*^k^*. Consequently, we can obtain

(6) $$\mathrm{Pr}[\mathrm{ReEnc}\_\mathrm{Err}]\;\leq \;\frac{n_{ren}}{2^{k}}$$

(7) $$\mathrm{Pr}[\mathrm{Dec}\_\mathrm{Err}]\;\leq \;\frac{n_{dec}}{2^{k}}.$$

When the above game is perfectly finished without any error, A has no better advantage in guessing *λ*. Therefore, it could be deduced that

Pr[*λ*′ = *λ* | SIP] = 1/2

⇒ Pr[*λ*′ = *λ* ∧ SIP] = 1/2Pr[SIP] ≤ Pr[*λ*′ = *λ*]

⇒ 1/2(1 − Pr[¬SIP]) − 1/2 ≤ Pr[*λ*′ = *λ*] − 1/2

⇒ −(1/2)Pr[¬SIP] ≤ *ε*

⇒ −(1/2)Pr[ReEnc_Err ∨ Dec_Err ∨ QH] ≤ *ε*

$$\Rightarrow \;\mathrm{Pr}[\mathrm{QH}]\;\geq \;2\varepsilon \;-\;\frac{n_{ren}+n_{dec}}{2^{k}}$$

When the event QH occurs, it can be learned that the *h*_2_-table would keep a new record of (*W*, *v*_2_), in which

$$W=a^{*{\left(sx^{*}\right)}^{-1}}=e(Y_{j}{}^{z^{*}},\;g^{t}{)}^{{\left(sx^{*}\right)}^{-1}}=e{\left(g^{z^{*}},\;g\right)}^{s^{-1}t}$$

Hence, B could solve the BIDHP by computing $W^{z{*}^{-1}}$. The non-negligible advantage of the BIDHP breaker B could be represented as *ε*′ ≥ (2*ε* − $\frac{n_{ren}+n_{dec}}{2^{k}}$)/*n_h_*__2__ and the execution time is bounded by *t*′ < *t* + *t_b_*(2*n_ren_* + 2*n_dec_* + 1), in which *t_b_* is the time to carry out a bilinear pairing operation.

### 4.3. Performance Evaluation

In a PRE scheme, the processes of encryption, re-encryption, and decryption are considered as major operations. Hence, we will compare the efficiency of these algorithms in the proposed scheme with a previous work addressed by Kim and Lee [36]. There are two schemes introduced in the literature [36]. One is a data management scheme based on PRE and the other is a data sharing scheme based on attribute PRE. The first mechanism introduced by Kim and Lee is closely related to the proposed scheme, since it also has the properties of multi-hop and constant-size ciphertext. In particular, this scheme is designed for IoT-based environments. To obtain a fair evaluation result, we only take their first scheme as a comparison.

Table 2 is the comparison of security and computation complexity. It is evident that the Kim–Lee scheme failed to provide provable security and the cryptographic assumption of their protocol is unknown too. As to the computation complexity, the proposed scheme outperforms theirs by two bilinear pairing operations. In the perspective of amount of data sharing, both schemes could utilize the proxy sever to share data among *n* IoT nodes, so as to reduce the encryption costs. That is, the amount of sharing for both schemes is O(*n*) as compared to the traditional way of O(*n*(*n* − 1)) without utilizing the proxy server.

For simplicity, the authors merely consider the above time-consuming computation, i.e., bilinear pairing and exponentiation. According to the research of Scott et al. [37], the two operations would separately take (2.97, 0.54) milliseconds on an Intel Pentium IV CPU of 3 GHz. Table 3 compares data sharing duration according to number of devices. We calculate the node count from 2 to 50. For instance, when the node count is 50, we could derive that the communication count for the traditional way is 50 * 49 = 2450 while that of the proposed and the Kim–Lee schemes is 50 * 1 = 50. Since the Re-Encrypt computation of the proposed scheme is only *T_E_*, which is better than that of Kim–Lee, i.e., *T_B_* + 2*T_E_*, we hence derive the computation duration with respect to various node counts as shown in Table 3.

**Figure 3:**
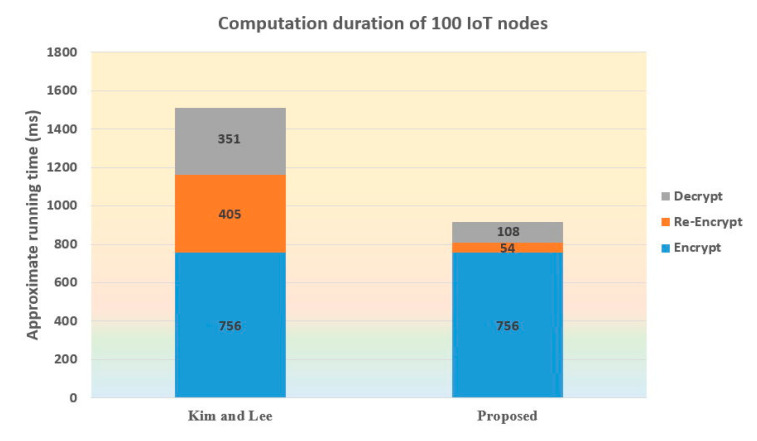
Comparison of computation duration of 100 IoT nodes.

Figure 3 shows the comparison of computation duration of 100 IoT nodes. It can be seen that the Kim–Lee scheme would take more time for the entire procedure (which is composed of Encrypt, Re-Encrypt, and Decrypt operations). Although the Encrypt algorithm of both schemes has identical running time, i.e., 756 ms, the Re-Encrypt and Decrypt algorithms of the Kim–Lee scheme obviously take much more time.

**Figure 4:**
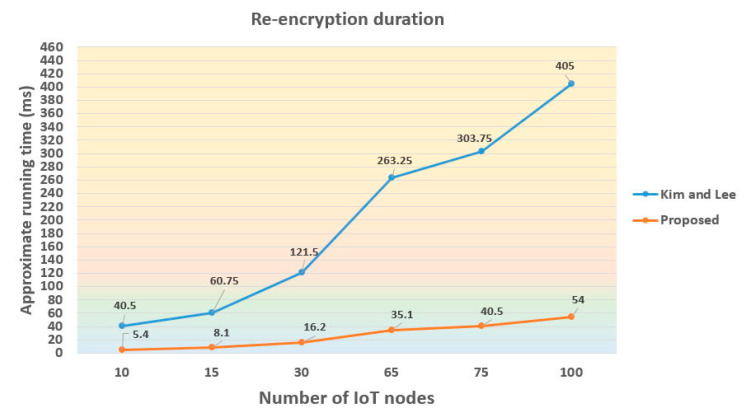
Comparison of re-encryption duration among different number of IoT nodes.

In the evaluation of re-encryption duration among 10 to 100 IoT nodes as illustrated in Figure 4, both the Kim–Lee and our scheme will spend more running time when the involved IoT nodes increase. Nevertheless, the re-encryption duration of theirs would grow much faster with the increased number of IoT nodes.

**Figure 5:**
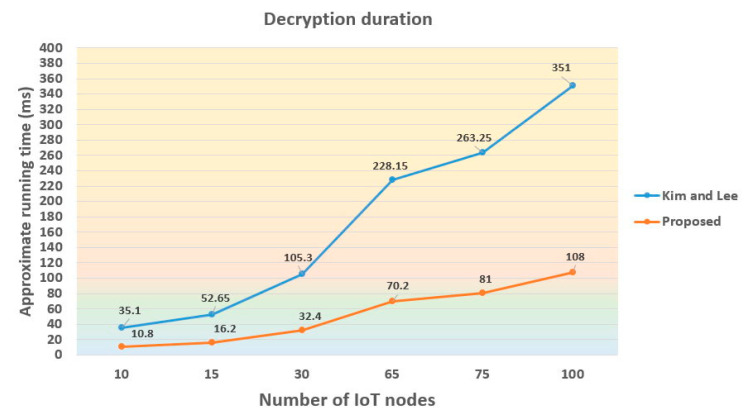
Comparison of decryption duration among different number of IoT nodes.

We demonstrate the decryption duration among different number of IoT nodes for the Kim–Lee and the proposed scheme in Figure 5. As shown in this figure, the duration curve of the former has a steeper slope than that of the latter. Take the case of 30 IoT nodes as an example, the proposed method would outperform the Kim–Lee one by 72.9 ms. In particular, the gap of duration dramatically enlarges according to the added IoT node count.

### 4.4. Discussion of Results

In the proposed PRE scheme, we attempt to reduce the computational complexity of designed algorithms. As indicated in Table 2, the total computational cost of our mechanism is 2*T_B_* + 6*T_E_*. In particular, we optimize the algorithms of ReEncrypt and Decrypt which are both pairing-free. On the contrary, these two algorithms of the Kim–Lee scheme have to take at least one pairing computation, which will inevitably incur higher computation and communication overheads when a large number of IoT nodes is involved in the system. The simulation results showed in Table 3 clearly reveal that the running time of the Re-Encrypt process alone in the Kim–Lee scheme has been longer than that of the proposed Re-Encrypt and Decrypt processes together. Although the simulated running time (as well as the computational efforts) of the Kim–Lee and the proposed schemes would naturally increase with the deployed number of IoT devices, illustrated in Figure 4 and Figure 5, the growing rate of the running time curve in the proposed system is apparently lower.

## 5. Conclusions

To improve the security of gradually popular IoT-based data outsourcing services in clouds, in this paper, we came up with an efficient proxy re-encryption scheme with constant-size ciphertext. In particular, our scheme is bidirectional and supports the functionality of multi-hop, which enables a proxy server to transform the ciphertext multiple times. A significant property of the proposed mechanism is that the re-encryption process only requires one exponentiation computation. Using the cryptographic assumption of intractable BIDHP, the proposed PRE scheme could withstand the adaptive-chosen ciphertext attacks in the security proof model of random oracles. We also demonstrate that our mechanism exhibits better efficiency than a related protocol introduced by Kim and Lee. Specifically, the computation complexity of the proposed method is 2*T_B_* + 6*T_E_* which takes approximately 918 ms running time when sharing data with 100 IoT devices. The simulation results in Figure 5 also reveal that the decryption duration of our approach only requires 32.4 ms in the communication environment of 30 IoT nodes. The future work will incorporate more superior functionalities (such as hierarchical access control) with existing PRE schemes to fulfill more comprehensive application requirements.

## Figures and Tables

**Table 1:** Definition of parameters.

Parameter	Description
*k*	security parameter
*q*	large prime
***G***_1_, ***G***_2_	multiplicative group
*g*	generator
*e*	bilinear map
*h*_1_, *h*_2_	one-way hash function
*x_i_*	private key
*Y_i_*	public key
*rk_i_*_→_ *_j_*	re-encryption key
*m*	message
*δ*	ciphertext

**Table 2:** Comparison of the proposed scheme.

	Scheme	Kim and Lee	Proposed
Item			
**Provable Security**		X	√
**Cryptographic Assumption**		Unknown	BIDHP
**Public Information**		{*G*, *G_T_*, *e*, *g*, *q*, *H*}	{*G*_1_, *G*_2_, *e*, *g*, *q*, *h*_1_, *h*_2_}
**Computation Complexity**		4*T_B_* + 6*T_E_*	2*T_B_* + 6*T_E_*
Amount of Sharing *		O(*n*)	O(*n*)

* Remark: The symbol *n* is the number of data-sharing nodes while (*T_B_*, *T_E_*) separately denotes the time for bilinear pairing and exponentiation.

**Table 3:** Data sharing duration according to number of devices.

NodeCount	Traditional Way		Kim and Lee		Proposed	
	Comm. Count	Duration (ms)	Comm. Count	Duration (ms)	Comm. Count	Duration (ms)
**2**	2	8.10	2	8.10	2	1.08
**3**	6	24.3	3	12.15	3	1.62
**4**	12	48.6	4	16.2	4	2.16
**5**	20	81	5	20.25	5	2.7
**-**	-	-	-	-	-	-
**50**	2450	9922.5	50	202.5	50	27

## References

[1] Wang C., Dong S., Zhao X., Papanastasiou G., Zhang H., Yang G. (2020). SaliencyGAN: Deep learning semisupervised salient object detection in the fog of IoT. IEEE Trans. Ind. Inf..

[2] Sidhu M.S., Saif S., Ghazali N.E., Shah S.M., Chun T.W., Hussain T.J. Automating switchgear asset supply chain management with IoT and RFID technology. Proceedings of the 2020 8th International Conference on Information Technology and Multimedia (ICIMU).

[3] Minerva R., Biru A., Rotondi D. (2015). Towards a Definition of the Internet of Things (IoT). https://iot.ieee.org/images/files/pdf/IEEE_IoT_Towards_Definition_Internet_of_Things_Revision1_27MAY15.pdf.

[4] Sethi P., Sarangi S.R. (2017). Internet of things: Architectures, protocols, and applications. J. Electr. Comput. Eng..

[5] Divarcı S., Urhan O. Secure gateway for network layer safety in IoT systems. Proceedings of the 2018 26th Signal Processing and Communications Applications Conference (SIU).

[6] Yassein M.B., Shatnawi M.Q., Al-zoubi D. Application layer protocols for the Internet of things: A survey. Proceedings of the 2016 International Conference on Engineering & MIS (ICEMIS).

[7] Tschofenig H., Arkko J., Thaler D., McPherson D. (2020). Architectural Considerations in Smart Object Networking.

[8] Chunpeng G., Liu Z., Xia J., Liming F. (2020). Revocable identity-based broadcast proxy re-encryption for data sharing in clouds. IEEE Trans. Dependable Secure Comput..

[9] Rawal B.S. A proxy re-encryption-based webmail and file sharing system for collaboration in cloud computing environment. Proceedings of the 2018 International Conference on Computational Techniques, Electronics and Mechanical Systems (CTEMS).

[10] Xu P., Jiao T., Wu Q., Wang W., Jin H. (2016). Conditional identity-based broadcast proxy re-encryption and its application to cloud email. IEEE Trans. Comput..

[11] Kanchan S., Chaudhari N.S. Integrating group signature scheme with non-transitive proxy re-encryption in VANET. Proceedings of the 2016 International Conference on Computing, Analytics and Security Trends (CAST).

[12] Wang X.A., Xhafa F., Zheng Z., Nie J. Identity based proxy re-encryption scheme (IBPRE+) for secure cloud data sharing. Proceedings of the 2016 International Conference on Intelligent Networking and Collaborative Systems (INCoS).

[13] Wang X.A., Xhafa F., Hao W., He W. Non-transferable unidirectional proxy re-encryption scheme for secure social cloud storage sharing. Proceedings of the 2016 International Conference on Intelligent Networking and Collaborative Systems (INCoS).

[14] Henriques M.S., Vernekar N.K. Using symmetric and asymmetric cryptography to secure communication between devices in IoT. Proceedings of the 2017 International Conference on IoT and Application (ICIOT).

[15] Li Z., Ma C., Wang D. (2017). Towards multi-hop homomorphic identity-based proxy re-encryption via branching program. IEEE Access.

[16] Ge C., Susilo W., Wang J., Huang Z., Fang L., Ren Y. (2016). A key-policy attribute-based proxy re-encryption without random oracles. Comput. J..

[17] Fugkeaw S., Sato H. Improved lightweight proxy re-encryption for flexible and scalable mobile revocation management in cloud computing. Proceedings of the 2016 IEEE 9th International Conference on Cloud Computing (CLOUD).

[18] Chandu Y., Kumar K.S.R., Prabhukhanolkar N.V., Anish A.N., Rawal S. Design and implementation of hybrid encryption for security of IoT data. Proceedings of the 2017 International Conference on Smart Technologies for Smart Nation (SmartTechCon).

[19] Fang L., Susilo W., Ge C., Wang J. (2012). Chosen-ciphertext secure anonymous conditional proxy re-encryption with keyword search. Theor. Comput. Sci..

[20] Wang X.A., Huang X., Yang X., Liu L., Wu X. (2012). Further observation on proxy re-encryption with keyword search. J. Syst. Softw..

[21] Liang K., Fang L., Susilo W., Wong D.S. A ciphertext-policy attribute-based proxy re-encryption with chosen-ciphertext security. Proceedings of the IEEE 2013 5th International Conference on Intelligent Networking and Collaborative Systems (INCoS), Xi’an.

[22] Akhil N.V., Vijay A., Kumar D.S. QR code security using proxy re-encryption. Proceedings of the 2016 International Conference on Circuit, Power and Computing Technologies (ICCPCT).

[23] Zeng P., Choo K.R. (2018). A new kind of conditional proxy re-encryption for secure cloud storage. IEEE Access.

[24] Hussain I., Negi M.C., Pandey N. Proposing an encryption/decryption scheme for IoT communications using binary-bit sequence and multistage encryption. Proceedings of the 2018 7th International Conference on Reliability, Infocom Technologies and Optimization (Trends and Future Directions) (ICRITO).

[25] Krishnamoorthy S., Muthukumaran V., Yu J., Balamurugan B. A secure privacy preserving proxy re-encryption scheme for IoT security using near-ring. Proceedings of the 2019 International Conference on Pattern Recognition and Artificial Intelligence.

[26] Fan C.I., Tseng Y.F., Huang Y.L. Key-aggregate proxy re-encryption with dynamic condition generation using multilinear map. Proceedings of the 2020 15th Asia Joint Conference on Information Security (AsiaJCIS).

[27] Chandrakala B.M., Reddy S.C.L. Proxy re-encryption using MLBC (modified lattice based cryptography). Proceedings of the 2019 International Conference on Recent Advances in Energy-efficient Computing and Communication (ICRAECC).

[28] Fimiani G. Supporting privacy in a cloud-based health information system by means of fuzzy conditional identity-based proxy re-encryption (FCI-PRE). Proceedings of the 2018 32nd International Conference on Advanced Information Networking and Applications Workshops (WAINA).

[29] Lian Z., Su M., Fu A., Wang H., Zhou C. Proxy re-encryption scheme for complicated access control factors description in hybrid cloud. Proceedings of the 2020 IEEE International Conference on Communications (ICC).

[30] Maiti S., Misra S. (2020). P2B: Privacy preserving identity-based broadcast proxy re-encryption. IEEE Trans. Veh. Technol..

[31] Meiliasari R.P., Syalim A., Yazid S. Performance analysis of the symmetric proxy re-encryption scheme. Proceedings of the 2019 International Workshop on Big Data and Information Security (IWBIS).

[32] Rabieh K., Mercan S., Akkaya K., Baboolal V., Aygun R.S. Privacy-preserving and efficient sharing of drone videos in public safety scenarios using proxy re-encryption. Proceedings of the 2020 IEEE 21st International Conference on Information Reuse and Integration for Data Science (IRI).

[33] Seo J.W., Yum D.H., Lee P.J. (2013). Proxy-invisible CCA-secure type-based proxy re-encryption without random oracles. Theor. Comput. Sci..

[34] Wu L., Yang X., Zhang M., Liu L. (2019). New identity based proxy re-encryption scheme from lattices. China Commun..

[35] Shen Y., Zhang H., Fan Y., Lee A.P.W., Xu L. (2020). Smart health of ultrasound telemedicine based on deeply-represented semantic segmentation. IEEE Internet Things J..

[36] Kim S., Lee I. (2018). IoT device security based on proxy re-encryption. J. Ambient Intell. Humaniz. Comput..

[37] Scott M., Costigan N., Abdulwahab W. Implementing cryptographic pairings on smartcards. Proceedings of the Workshop on Cryptographic Hardware and Embedded Systems 2006 (CHES 2006).

